# Supplementary material for: Validation of the FACETS-OF-PPC as an Outcome Measure for Children with Severe Neurological Impairment and Their Families—A Multicenter Prospective Longitudinal Study
Source: Children (Basel). 2021 Oct 11;8(10):905. doi: 10.3390/children8100905 (PMC8535047; doi:10.3390/children8100905)
Supplement: Supplementary file 1 [file children-08-00905-s001.zip › children-1386535-supplementary.pdf]

FACETS-OF-PPC Scales and Corresponding Items

Original Model

Scale	Items
Symptoms	B1 + B2 + B3 + B4 + B5 + B6 + B7 + C10
Child's social participation	A3 + A4 + C1 + C2
Normalcy	C4 + C5 + C6 + C8 + C11 + C12
Social support	C3 + C7 + C9
Coping with the disease	A5 + A10 + A13 + A14
Caregiver's competencies	A1 + A2 + A6 + A7 + A8 + A9 + A11 + A12 + A15

Revised Model 1

Scale	Items
Child's social participation	A3 + A4 + C1 + C2
Normalcy	C4 + C5 + C6 + C8 + C11 + C12
Social support	C3 + C7 + C9
Coping with the disease	A5 + A10 + A13 + A14
Caregiver's competencies	A1 + A2 + A6 + A7 + A8 + A9 + A11 + A12 + A15

Revised Model 2

Scale	Items
Child's social participation	A3 + A4 + C1
Normalcy	C4 + C5 + C6 + C8 + C12
Social support	C3 + C7 + C9
Coping with the disease	A5 + A10 + A14
Caregiver's competencies	A6 + A7 + A8

Item Deletions

Step	Item	Scale deleted from
1	All B and C10	Symptoms
2	A13	Coping with the disease
3	C2	Child's social participation
4	A15	Caregiver's competencies
5	C11	Normalcy
6	A2	Caregiver's competencies
7	A12	Caregiver's competencies
8	A11	Caregiver's competencies
9	A9	Caregiver's competencies
